# Treatment of gastric peritoneal carcinomatosis by combining complete surgical resection of lesions and intraperitoneal immunotherapy using catumaxomab

**DOI:** 10.1186/1471-2407-14-148

**Published:** 2014-03-04

**Authors:** Diane Goéré, Nathalie Gras-Chaput, Anne Aupérin, Caroline Flament, Christophe Mariette, Olivier Glehen, Laurence Zitvogel, Dominique Elias

**Affiliations:** 1 Department of Surgical Oncology - Gustave Roussy, 114 rue Edouard Vaillant, Villejuif, Cedex 94805, France; 2 Institut National de la Santé et de la Recherche Médicale, U1015, Gustave Roussy, 114 rue Edouard Vaillant, Villejuif, Cedex 94805, France; 3 Department of Statistics - Gustave Roussy, 114 rue Edouard Vaillant, Villejuif, Cedex 94805, France; 4 Department of Digestive and Oncological Surgery, University Hospital C. Huriez, Place de Verdun, Lille, Cedex 59037, France; 5 Department of Surgical Oncology, Centre Hospitalier Lyon Sud, Pierre Bénite 69495, France

**Keywords:** Peritoneal carcinomatosis, Gastric carcinoma, Intraperitoneal chemotherapy, Immunotherapy, Catumaxomab

## Abstract

**Background:**

The peritoneum is one of the most frequent sites of recurrent gastric carcinoma after curative treatment, despite the administration of pre- and/or postoperative systemic chemotherapy. Indeed, the prognosis of peritoneal carcinomatosis from gastric carcinoma continues to be poor, with a median survival of less than one year with systemic chemotherapy. Whereas the prognosis of peritoneal carcinomatosis from colorectal cancer has changed with the development of locally administered hyperthermic intraperitoneal chemotherapy (HIPEC), survival results following carcinomatosis from gastric cancer remain disappointing, yielding a 5-year survival rate of less than 20%. Innovative surgical therapies such as intraperitoneal immunotherapy therefore need to be developed for the immediate postoperative period after complete cytoreductive surgery. In a recent randomised study, a clinical effect was obtained after intraperitoneal infusion of catumaxomab in patients with malignant ascites, notably from gastric carcinoma. Catumaxomab, a nonhumanized chimeric antibody, is characterized by its unique ability to bind to three different types of cells: tumour cells expressing the epithelial cell adhesion molecule (EpCAM), T lymphocytes (CD3) and also accessory cells (Fcγ receptor). Because the peritoneum is an immunocompetent organ and up to 90% of gastric carcinomas express EpCAM, intraperitoneal infusion of catumaxomab after complete resection of all macroscopic disease (as defined in the treatment of carcinomatosis from colorectal cancer) could therefore efficiently treat microscopic residual disease.

**Methods/design:**

The aim of this randomized phase II study is to assess 2-year overall survival after complete resection of limited carcinomatosis synchronous with gastric carcinoma, followed by an intraperitoneal infusion of catumaxomab with different total doses administered in each of the 2 arms. Close monitoring of peri-opertive mortality, morbidity and early surgical re-intervention will be done with stopping rules. Besides this analysis, translational research will be conducted to determine immunological markers of catumaxomab efficacy and to correlate these markers with clinical efficacy.

## Background

### Rationale

#### Peritoneal carcinomatosis from gastric carcinoma

Although the incidence of gastric cancer has decreased during the past years, it is still the fourth most common newly diagnosed cancer worldwide and the second leading cause of cancer-related death [1]. Surgery comprising a subtotal or total gastrectomy with a D1.5-D2 lymphadenectomy, remains the most important tool for curative treatment of gastric carcinoma. Surgery is usually combined with systemic perioperative chemotherapy whose benefit has been demonstrated in two randomized studies: the MAGIC trial [2] using epirubicin, cisplatin, and a continuous infusion FU regimen and the FNLCC-FFCD trial [3] using 5-FU and cisplatin. In the latter trial, 5-year overall survival was significantly longer in patients in the perioperative chemotherapy group compared to those in the surgery alone group, respectively 38% and 24%.

In addition, despite the administration of pre- and/or postoperative systemic chemotherapy, one of the major problems with gastric carcinoma is its peritoneal tropism and the peritoneum is the major site of recurrence. Peritoneal dissemination commonly occurs in patients with gastric cancer via intracoelomic dissemination or due to tumour spillage during surgery [4]. Peritoneal carcinomatosis is present at the diagnosis in 5-20% of the patients [5] and can affect 60% of the patients after curative treatment [6], and this tumour manifestation is considered a fatal disease with limited treatment options. Thus, in the multicentric prospective study of peritoneal carcinomatosis (EVOCAPE 1 study), median overall survival for the natural history of the disease was 3.1 months [7].

#### Intraperitoneal chemotherapy for gastric carcinoma

A new therapeutic approach for peritoneal carcinomatosis has been under development for over twenty years. This treatment consists of complete cytoreductive surgery of peritoneal lesions followed by intraperitoneal chemotherapy. The main objective of the intraperitoneal administration of chemotherapy is to heighten the concentration and the total amount of the drug, thereby reducing plasma concentrations. This treatment increased the survival of patients with carcinomatosis of colorectal origin, from pseudomyxoma and mesothelioma [8-10]. Regarding carcinomatosis of gastric origin, the results of intraperitoneal chemotherapy with conventional chemotherapeutic agents such as mitomycin C, oxaliplatin and 5FU remain disappointing. In a selected population of 159 patients with peritoneal disease alone, cytoreductive surgery plus intraperitoneal chemotherapy yielded a 5-year overall survival rate of 13% and a median overall survival rate of only 9.2 months [11]. However, the beneficial effect of hyperthermic intraperitoneal chemotherapy (HIPEC) in the treatment of PC from gastric carcinoma was demonstrated in a phase III trial [12]. Sixty-eight patients with gastric PC were randomized between complete cytoreductive surgery alone (n = 34) or complete cytoreductive surgery followed by HIPEC (n = 34) with cisplatin (120 mg) and mitomycin C (30 mg) each in 6000 ml of normal saline at 43°C over 60-90 min. Macroscopic complete cytoreduction of PC was achieved in 58.8% of the patients in each arm. Median survival was significantly increased in the group who received HIPEC, but it was only 11 months (95% confidence interval 10-11.9 months), compared to 6.5 months (95% confidence interval 4.8-8.2 months) in the surgery alone group (P = 0.046). A multivariate analysis found that complete cytoreductive surgery plus HIPEC, synchronous PC, the completeness of surgery (0-1), systemic chemotherapy ≥ 6 cycles, and no serious adverse events were independent predictors for better survival.

Thus, because of the poor prognosis of PC of gastric origin and disappointing results of treatment with complete cytoreductive surgery followed by HIPEC containing cytotoxic agents, innovative surgical therapies such as intraperitoneal immunotherapy need to be developed for the immediate postoperative period after complete cytoreductive surgery.

#### Intraperitoneal immunotherapy with catumaxomab in malignant ascites

Catumaxomab is a nonhumanised chimeric antibody, consisting in a mouse-derived anti-EpCAM Fab (fragment antigen-binding) region and a rat anti-CD3 Fab. Thus, catumaxomab is characterized by its unique ability to bind to three different types of cells: tumor cells expressing the epithelial cell adhesion molecule (EpCAM positive), T lymphocytes (CD3 positive) and also accessory cells (Fcγ receptor positive), such as macrophages, natural killer cells and dendritic cells, which are activated with the hybrid Fc (crystallisable fragment) region [13,14]).

Catumaxomab anti-tumour activity has been demonstrated in vitro, notably in ascitic fluids, resulting in a decreased rate of EpCAM + cells and the release of pro-inflammatory cytokines (Interferon- γ, tumour necrosis factor-α, interleukin (IL)-2 and IL-6) [15].

A randomized study was performed in patients with symptomatic malignant ascites secondary to EpCAM + carcinomas, to evaluate the efficacy and safety of intraperitoneal administration of catumaxomab [16]. Patients were randomly assigned to paracentesis alone, or to paracentesis plus intraperitoneal catumaxomab. The efficacy of intraperitoneal administration of catumaxomab was evaluated on puncture-free survival (primary endpoint). Two hundred and fifty-eight patients, 66 of whom had carcinomatosis of gastric origin, were included in the study. Catumaxomab was administered in four intraperitoneal infusions, each preceded by aspiration of ascites at day 0 (D0), D3, D7 and D10, and required a total of 11 days of hospitalization. Puncture-free survival was significantly longer in the group treated with catumaxomab compared to that in the control group (46 *vs* 11 days, p < 0.0001). Median overall survival was similar between the two groups: 72 days in the catumaxomab group *vs* 68 days in the control group. The most common adverse events were related to the release of cytokines (fever, nausea, vomiting, tachycardia and hypotension). These reactions were of mild or moderate severity (grade 1 or 2) and transient, limited to the duration of catumaxomab therapy with an acceptable tolerability profile. Other treatment-related adverse events were haematological (lymphopenia, leucocytosis and anaemia) and non-haematological (abdominal pain, elevated C-reactive protein, and gamma-glutamyltransferase levels, fatigue, anorexia, elevated blood alkaline phosphatase, AST and ALT levels). This study confirmed the feasibility and efficacy of intraperitoneal infusion of catumaxomab in reducing the volume of ascites. Thus, an approval was granted to the European Union in April 2009 for the use of catumaxomab intraperitoneal infusion in patients with ascites from an EpCAM + malignancy for which standard therapy was not available or no longer feasible [17]. This study also demonstrated that deterioration in quality of life (QoL) scores, evaluated with the EORTC QLQ-C30, appeared significantly more rapidly in the control than in the catumaxomab group for all scores (range of median times 19-26 days *vs* 47-49 days, p < 0.0001) [18].

Another randomized study was reported at ASCO congress in 2008 [19]. Among 55 patients operated on for gastric adenocarcinoma (T2b/T3/T4, N±, M0) with a curative intent, 28 received an intraperitoneal catumaxomab infusion during the immediate postoperative period, and were compared to 27 patients who underwent resection alone. Catumaxomab was administered during surgery and then 4 times (D7, 10, 13 and 16) at increasing doses. The EpCAM antigen was present in 100% of patients. Seventy-eight percent (22/28) of the patients treated with catumaxomab received all 5 infusions. Treatment-related adverse events occurred in 40% of the patients (grade 3 in 22 patients, of whom 10 were in control arm). The most frequent adverse events in the catumaxomab group were anaemia, pyrexia, inflammatory syndrome and abdominal pain. All related serious adverse events resolved at the end of therapy except for nephropathy (one patient), which resolved leaving minor sequels. This study demonstrated that adjuvant intraperitoneal administration of catumaxomab, after gastrectomy, seems to be feasible, safe and well tolerated.

A *post hoc* analysis was performed to investigate whether there was a correlation between the detection of human anti-mouse antibodies (HAMAs) 8 days after the fourth catumaxomab infusion and clinical outcome, because catumoxamab is a mouse/rat antibody [20]. Among patients who received intraperitoneal catumaxomab, those who developed HAMAs experienced greater clinical benefits (longer puncture-free survival, longer time to the next therapeutic puncture) than those who did not develop HAMAs. In addition, median survival was significantly longer in the group of patients who developed HAMAs (129 vs 64 days, p = 0.0003; hazard ratio 0.433). These results demonstrate that there was a strong correlation between humoral immune response to catumaxomab and clinical outcome in that phase II/III study.

However, the survival benefit of immediate postoperative immunotherapy after cytoreductive surgery of peritoneal carcinomatosis has never been reported. There are strong arguments to evaluate this treatment in patients with gastric carcinomatosis: [1] the poor prognosis of patients despite optimal treatment with a curative intent including HIPEC [2], the expression of the EpCAM antigen in nearly 90% of gastric adenocarcinoma [21], and [3] peritoneum is an immunocompetent organ, [4] peritoneal mesenchymal cells do not express the EpCAM antigen.

Due to our experience in the surgical treatment of peritoneal carcinomatosis which represents a major activity of the Department of Surgical Oncology (over 700 patients since 1993), we are poised to initiate a phase II randomised clinical trial (IIPOP), funded by a PHRC grant (Programme Hospitalier de Recherche Clinique).

## Methods/design

The IIPOP study is a multicentre, open-label, phase II randomised study. The investigator initiated trial (IIT) will be conducted by the Department of Surgery of the Gustave Roussy Institute (Villejuif, France) in collaboration with the University Hospital C. Huriez (Lille, France) and the University Hospital Lyon Sud (Pierre Bénite, France).

### Study objectives and endpoints

The primary objective of the IIPOP study in patients with synchronous and limited PC arising from gastric carcinoma, is to estimate 2-year overall survival (OS). Each treatment arm will be compared to theoretical rates using a design with one single stage (no interim analysis of the primary criterion of efficacy).

The secondary endpoints are:

– morbidity and toxicity: postoperative mortality (D30 or until discharge from hospital), acute toxicity (D30 or until discharge from hospital) according to the NCI-CTC-AE toxicity scale version 4.0 [22], and side effects more specifically associated with catumaxomab: systemic inflammatory syndrome (SIRS), hepatotoxicity, skin reaction of an allergic type (temperature, level of haemoglobin, hepatic profile (ALAT, ASAT, alkaline phosphatases, bilirubin) on D3, D6 and D9),

– the 2-year peritoneal relapse rate,

– the 2-year relapse-free survival rate,

– 5-year overall survival.

Translational research based on an immunological analysis will be performed, to determine the immunological markers of the efficacy of catumaxomab, to correlate these markers with clinical efficacy.

### Study population

The study population of the IIPOP study will comprise patients with synchronous and limited PC arising from histologically proven gastric carcinoma, confirmed by a frozen section histological examination during surgery.

The extent of peritoneal tumour spread (Peritoneal cancer Index, PCI [23]) calculated during surgery, must be equal to or lower than 12 and amenable to complete resection. Resection of all visible lesions (primary, lymph nodes, carcinomatosis) must be performed before randomisation, meaning that there should be no residual deposit exceeding 1 mm in diameter. Moreover, patients to be included in the study must fulfil the following inclusion criteria: age over 18 years, a good general health status (WHO 0–1), absence of haematogenous metastases, a signed consent form before surgery, no pregnancy or breast feeding, adequate contraception in fertile patients and adequate private or national insurance coverage.

Exclusion criteria include: previous treatment with a non-humanised (mouse or rat) monoclonal antibody, a known allergy to murine or chimeric monoclonal antibodies, another malignancy than the disease under study or a second cancer < 5 years earlier, inclusion in other clinical trials, absence of any psychological, familial, sociological or geographical condition potentially hampering compliance with the study protocol and follow-up. Patients could have received systemic chemotherapy before surgery.

### Treatment schedule (Figure 1)

Patients will receive intraperitoneal catumaxomab after complete resection of intra-abdominal lesions. The original concept of this study is to undertake the initial treatment combining surgery treating the visible disease (macroscopic) and intraperitoneal immunotherapy treating the remaining invisible (microscopic) disease. The latter will be administered via a continuous peritoneal infusion over 6 days postoperatively with 3 injections of catumaxomab every 48 hours at increasing doses in order to limit systemic inflammatory reactions.

First, surgery will consist of the resection of the primary (gastrectomy plus D1.5 lymphadenectomy) and of all peritoneal deposits (no residual disease greater than 1 mm). At the end of the procedure, three 20 or 25 French tubular drains will be placed, one under each of the diaphragmatic cupolas and one in Douglas’ pouch, to infuse the dialysate containing catumaxomab.

Second, the continuous intraperitoneal immunotherapy with catumaxomab (sponsored by Fresenius®) will be administered immediately after surgery to treat the microscopic residual tumour disease. The peritoneal volume will be increased (to 700 ml/m^2^) in order to bathe the entire abdominal cavity over a longer period more successfully than could be achieved with a lower volume, and it is adapted to each patient. Then the 3 drains will be clamped for 47 h, then unclamped for 1 hour. The second and third intraperitoneal infusion, will be delivered over a 3 h period by the inlet drain then the drains will be clamped for 44 h for the inlet drain and 47 h for the exit drains, then unclamped for 1 hour. Finally, the drains will be definitively unclamped on D6, and removed upon request.

**Figure 1:**
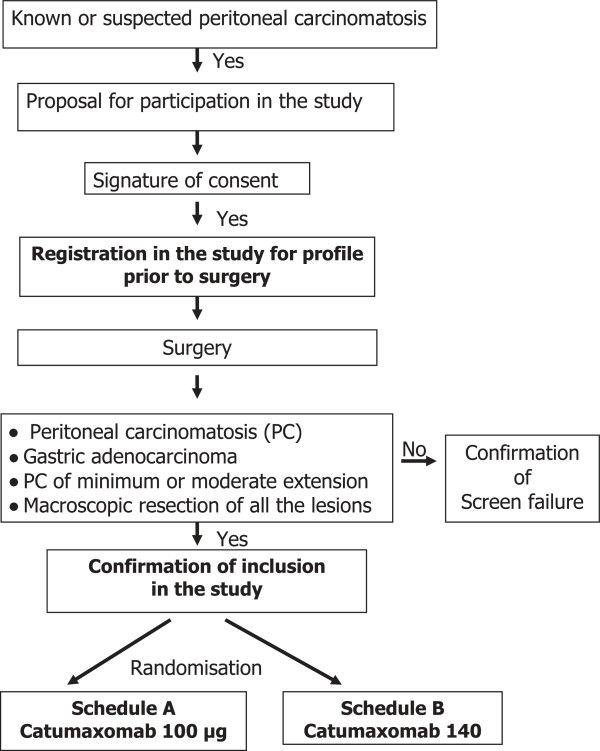
Study design.

The dosage of intraperitonal immunotherapy will be randomly allocated (Figure 1):

#### **Regimen A (100 μg):**

– on D0, 10 μg of catumaxomab over 3 hours in 250 ml of NaCl 0.9%, added to 700 ml/m^2^ of NaCl 0.9% immediately at the end of surgery

– on D2, 30 μg of catumaxomab in the same volume (700 ml/m^2^) over 3 hours using the same method, just after the 1-hour duration of unclamped drainage

– on D4, 60 μg of catumaxomab using the same method, just after the 1 hour-duration of unclamped drainage.

#### **Regimen B (140 μg):**

– on D0, 20 μg of catumaxomab over 3 hours in 250 ml of NaCl 0.9%, added to 700 ml/m^2^ of NaCl 0.9%, immediately at the end of surgery

– on D2, 40 μg of catumaxomab in the same volume (700 ml/m^2^) over 3 hours using the same method, just after the 1-hour duration of unclamped drainage

– on D4, 80 μg of catumaxomab using the same method, just after the 1-hour duration of unclamped drainage.

The randomization is stratified for center.

This treatment will be administered and monitored in the intensive care unit during the first 6 postoperative days.

### Early interruption of treatment

Treatment with catumaxomab should be halted in the following cases:

– Unexplained fever exceeding 39° for over 48 h

– Any serious complication requiring early surgical reintervention

– Any organ failure lasting longer than 48 h remaining unexplained

Any serious unexpected event will be discussed in a multidisciplinary meeting in the intensive care unit with surgeons and may result in the discontinuation of catumaxomab. Early interruption of the catumaxomab bath will be definitive.

### Assessments and follow-up

During the screening period, patients will be assessed for their eligibility to be included in the IIPOP study. Inclusion and exclusion criteria will be assessed by the investigator.

Morbidity and toxicity (D30 or until discharge from hospital) will be evaluated during the intraperitoneal treatment and the postoperative period. The severity of complications (Grade I-V) [24] will be assessed and adverse events will be categorized using the CTCAE version 4.0 [22].

The planned duration of follow-up is 5 years, with three-monthly follow-up visits consisting of a physical examination, laboratory tests (including tumour marker determination) and imaging (alternating between chest and abdomino-pelvic CT scan and abdominal ultrasound), over the first 2 years. From 2 to 3 years, monitoring will be done every 4 months, and after 3 years, every 6 months.

For the immunological monitoring, samples of peritoneal fluid will be collected at the beginning of the surgery, at the end of the procedure and during IP treatment on D2 and D6. Analysis of cellular immunity (qualification and quantification of cells (T cells, NK cells, macrophages, dendritic cells), of the cytokine profile and of the cytotoxicity (cell death) will be done on peritoneal fluids. Blood samples will be collected at the beginning of the surgery, just before discharge of hospital and 4 +/-1 months later, to study the innate and specific immunological response and the cytokine profile.

### Statistical considerations

#### Required number of patients

IIPOP is a phase II randomised trial. Each treatment arm will be compared to theoretical rates using a design with one single stage (no interim analysis of the primary criterion of efficacy). The 2-year survival rate after complete cytoreduction surgery and intraperitoneal chemotherapy is approximately 20%. Given the severity of the proposed treatment, a 2-year survival rate equal to or lower than 30% will be considered as unacceptable, and a 2-year survival rate equal to or higher than 55% will be considered as a promising survival rate. Forty randomised patients are required (20 in each arm). If 9 patients or more are still alive at 2 years, the treatment will be considered promising. If 8 or fewer patients are still alive at 2 years, treatment will not be considered sufficiently effective. Type I error (one-sided) is set at 11% and type II error is set at 13%. In order to randomise 40 patients, more patients must be enrolled. In fact, some patients will be considered ineligible at the end of surgery if at least one of the peroperative eligibility criteria is not met: no histological confirmation of gastric carcinoma, with peritoneal cancer index > 12 or no complete resection of the macroscopic lesions or no D2 or D1.5 lymphadenectomy. As approximately 1/3 of the patients operated on are expected to be eligible for randomisation, around 120 patients should be enrolled in order to randomise 40.

#### Statistical analysis plan

The main analysis of efficacy in each treatment arm will be carried out once all the patients in the said arm have been followed up for 2 years. Overall survival is defined as the period between the date of surgery and the date of death for whatever reason or the date of the latest news for patients who are still alive. Relapse-free survival is defined as the period between the date of surgery and the date of the first event (relapse or death for whatever reason) or the date of the latest news for patients who are still live and are free of recurrence. The time to a peritoneal relapse is defined as the time between the date of surgery and the date of the peritoneal relapse. Patients who die but were free of any peritoneal relapse at the time of death will be censored on the date of death. Patients who are still alive without a peritoneal relapse at the time of the most recent news will be censored on the date of the latest news. Overall survival, relapse-free survival and peritoneal relapse rates will be estimated using the Kaplan-Meier method. The rates at 2 years and 5 years will be estimated with their 95% confidence interval using the Rothman method. If, at the end of the trial, both treatment arms are considered to be promising, their efficacy and toxicity will be compared. However, as the trial was not planned to perform these comparisons, the power of these comparisons will be low and they may only be considered as exploratory analyses to be confirmed by subsequent studies.

The rates of grade 3-4 acute toxicity and the rates of early reinterventions as well as their 95% confidence intervals will be estimated in both treatment arms. This will also be carried out for the rate of the side effects more specifically associated with catumaxomab.

An intention to treat analysis will be conducted: all randomised subjects will be taken into account in the analysis depending on their randomisation group including those incorrectly included or those who do not meet protocol inclusion criteria. All the randomised patients will be analysed for the toxicity of the procedure. In order to be considered suitable for assessment of the toxicity of the intraperitoneal infusion of catumaxomab, patients must have received at least one injection.

The Biostatistics and Epidemiology Unit of the Institut Gustave Roussy is responsible for data management and analysis of this trial.

#### Toxicity monitoring

Sequential monitoring of mortality at 30 days (30D) will be performed in each treatment arm for each death (occurring before 30 days) once the second death has occurred. The 30D mortality rate post-surgery plus intraperitoneal chemotherapy was 6% in the recently published large French series [10]. Monitoring will be based on the one-sided exact binomial test at 10% of the following hypothesis: 30D mortality > 6%, without adjusting for multiple analyses which will be a maximum of 3 (no adjustment as a precaution). In the event of 2 deaths (before 30 days) occurring in 9 patients or fewer, or of 3 deaths (before 30 days) occurring in 18 patients or fewer, the treatment arm will be halted. If 4 deaths (before 30 days) are observed in the 20 patients included or fewer, mortality at 30 days will be considered too high and the treatment arm will be considered unacceptable.

The grade 3-4 toxicity rate at 30 days and the early surgical re-intervention rate at 30 days will also be evaluated in both treatment arms. The rate of grade 3-4 toxicity was 28% in the French surgery plus intraperitoneal chemotherapy series and the rate of early re-intervention was 26% [10]. An interim analysis will be performed on the first 10 patients in each arm and a final analysis on the 20 patients in each arm for each of these 2 endpoints. The analyses will be based on the one-sided exact binomial tests at 10% of the following hypotheses: for toxicity, rate of grade 3-4 > 30% and for early re-interventions, rate > 25%. For both endpoints, adjustment for the multiple analyses will be done using Pocock method.

### Quality assurance

The protocol will be conducted according to Good Clinical Practice (GCP) guidelines and to the ethical principles described in the Declaration of Helsinki. The study protocol was approved by the leading Ethics Committee and was subject to the approval of the national competent authority (ANSM, ref A120339-21) considered mandatory by federal law. The study was assigned the EudraCT number 2012-000475-174 and is registered at ClinicalTrials.gov (NCT01784900).

## Discussion

The peritoneum is one of the most frequent sites of recurrence from gastric carcinoma after curative treatment, despite the administration of pre- and/or postoperative systemic chemotherapy. In addition, the prognosis of peritoneal carcinomatosis from gastric carcinoma remains poor, and has not really been improved during recent years, despite the advent of targeted therapies. A meta-analysis reported that systemic chemotherapy extended median survival time to 11 months in patients with advanced gastric cancer compared to the best supportive care alone [25].

In an attempt to improve the prognosis of such patients operated on for gastric carcinoma, prophylactic HIPEC has been evaluated in patients at higher risk of developing peritoneal recurrence. A meta-analysis demonstrated that intraperitoneal chemotherapy combined with surgery yielded a positive effect on overall survival [26]. These results were confirmed in a recent large retrospective study of 360 patients with stage T2-4bN0-3 M0 gastric adenocarcinoma. It obtained a 5-year overall survival rate of 60.4% after surgery plus intraperitoneal chemotherapy compared to 42.9% when surgery alone was performed (p = 0.001) [27]. Whereas the prognosis of PC from colorectal cancer has changed with the development of locally administered HIPEC, survival results after HIPEC in the treatment of carcinomatosis from gastric cancer remain disappointing, yielding a 5-year survival rate of less than 20%.

Currently, there is no real effective treatment to offer to these patients. Innovative therapeutics are desperately required. The anti-tumour activity of catumaxomab has been demonstrated in vitro, in ascitic fluids from malignant ascites, notably from gastric carcinoma. A clinical effect on paracentesis-free survival and on the time to deterioration of QoL, has also been demonstrated in a randomised study [16]. Thus, the use of catumaxomab to treat microscopic residual disease after resection of peritoneal carcinomatosis from gastric carcinoma appears to be a good therapeutic option. The aim of the IIPOP randomized phase II study is to assess 2-year overall survival after complete resection of limited carcinomatosis (PCI ≤ 12) synchronous with gastric carcinoma, followed by intraperitoneal infusion of catumaxomab with different total doses administered in each of the 2 arms. Mortality, morbidity and specific toxicity will also be evaluated. In addition to these analyses, translational research will be conducted to determine immunological markers of the efficacy of catumaxomab and to correlate these markers with clinical efficacy.

## Abbreviations

PC: Peritoneal carcinomatosis; HIPEC: Hyperthermic intraperitoneal chemotherapy; Fab: Fragment antigen binding; EpCAM: Epithelial cell adhesion molecule; Fc: Fragment crystallisable region; IL: Interleukin; HAMAs: Human anti-mouse antibodies; SIRS: Systemic inflammatory syndrome; OS: Overall survival; ALAT: Alanine aminotransférase; ASAT: Aspartate aminotransférase; PCI: Peritoneal cancer index; CRF: Case report form.

## Competing interests

The IIPOP study is financially supported by the National Institute of Cancer (INCa), and by Fresenius for catumaxomab delivery. The authors have no competing interest to declare relative to this study.

## Authors’ contributions

DG: conception, data collection, data analysis, data interpretation, writing. NG-C: translational study design, data analysis. AA: statistical design and analysis. CF: translational study design, data analysis. CM: data collection, data analysis. OG: data collection, data analysis. LZ: translational study design, data analysis. DE: principal investigator, conception, data collection, data analysis, data interpretation, writing and final approval. All authors read and approved the final manuscript.

## Pre-publication history

The pre-publication history for this paper can be accessed here:

http://www.biomedcentral.com/1471-2407/14/148/prepub
